# Sequential patient–spouse dyadic psychological intervention for perioperative emotional distress and dyadic coping in patients with spinal metastases

**DOI:** 10.3389/fpubh.2026.1930615

**Published:** 2026-10-07

**Authors:** Liping Xie, Yunli Wang, Zhixiao Wang, Zeping Shao, Xiaoqing Han, Hui Xu, Dengxing Lun, Yating Wen, Yan Chen, Yingshu Yang, Baochang Ding, Xin Pan

**Affiliations:** 1 Weifang People’s Hospital, Weifang, China; 2 The 80th Group Army Hospital of the Chinese People’s Liberation Army (PLA), Weifang, China; 3 Weifang Eye Hospital, National Key Clinical Specialty, Zhengda Guangming Eye Group, Weifang, China

**Keywords:** spouse caregiver, caregiving burden, dyadic coping, dyadic intervention, emotional distress, perioperative nursing, spinal metastases

## Abstract

**Purpose:**

To evaluate the effects of a sequential patient–spouse dyadic psychological intervention on perioperative emotional distress, dyadic coping, and spouse caregiving burden in patients undergoing surgery for spinal metastases.

**Methods:**

Patients with spinal metastases undergoing surgical treatment and their spouses were recruited in this single-center, prospective randomized controlled trial. A total of 88 patient–spouse dyads were randomly assigned to the intervention group or the control group at a 1:1 ratio. Patient emotional distress measured using the Distress Thermometer (DT) was the primary outcome. Spouse DT, patient and spouse Dyadic Coping Inventory (DCI) scores, and spouse Zarit Burden Interview (ZBI) scores were secondary outcomes. T0 was treated as the baseline covariate, and postbaseline outcomes were analyzed using baseline-adjusted linear mixed-effects models. A supplementary dyad-aware mixed model jointly modeled patient and spouse outcomes to account for within-dyad dependence.

**Results:**

A total of 88 dyads (44 per group) were included in the analysis. For the primary outcome, patient DT scores were lower in the intervention group across the postoperative assessment period (adjusted average difference, −0.46; 95% CI, −0.79 to −0.12; Wald χ^2^ = 7.05; *p* = 0.008), although the patient-specific group-by-time interaction was not significant (*p* = 0.125). At 2 weeks after discharge, the adjusted patient DT difference was −0.71 (95% CI, −1.17 to −0.24; Holm-adjusted *p* = 0.009; Hedges’ g = −0.67, 95% CI, −1.10 to −0.24). Spouse DT scores were also lower overall (adjusted average difference, −0.62; 95% CI, −0.92 to −0.32; *p* < 0.001), with a standardized difference at 2 weeks of g = −0.79 (95% CI, −1.23 to −0.36). In the joint dyad-aware DT model, the group-by-time interaction was significant (Wald χ^2^ = 7.49, df = 2, *p* = 0.024), whereas the group-by-role-by-time interaction was not (*p* = 0.988). Patient and spouse DCI scores were higher overall in the intervention group (adjusted average differences, 5.50 and 3.33 points, respectively), but their role-specific group-by-time interactions were not significant. Spouse ZBI scores were lower overall in the intervention group (adjusted average difference, −3.24; 95% CI, −5.28 to −1.20; *p* = 0.002), without a significant group-by-time interaction.

**Conclusion:**

The sequential patient–spouse dyadic psychological intervention was associated with modestly lower emotional distress and higher dyadic coping during the later perioperative period. The findings were strongest for distress at the post-discharge assessment, but mean DT scores remained above the clinical screening threshold in both groups. These results support further evaluation of structured dyadic psychosocial care with longer follow-up and dyad-focused analyses rather than immediate broad implementation.

## 1 Introduction

Spinal metastasis is one of the common complications of advanced malignant tumors and can lead to pain, spinal instability, neurological impairment, and a marked decline in quality of life. With advances in multidisciplinary treatment, an increasing number of patients undergo surgery to relieve pain, improve or preserve neurological function, and enhance overall health status (1, 2). However, surgery does not eliminate the disease-related threats and psychological stress faced by these patients. A care model focusing solely on physical symptoms is no longer sufficient to meet their comprehensive care needs (3).

The perioperative period represents a critical stage during which psychological vulnerability may further increase in patients with spinal metastases. Before surgery, patients often experience concerns about surgical risks, persistent pain, functional deterioration, and disease progression. After surgery, pain control, physical weakness, uncertainty regarding recovery, and changes in life roles may further amplify their emotional burden. Studies involving patients undergoing surgery for metastatic spinal disease have found that nearly half of patients experience clinically significant distress before surgery, with pain, fatigue, and worry being the most frequently reported problems (2, 4, 5). Other studies have shown that depression is not uncommon during the perioperative period in patients with spinal metastases, and its burden tends to persist throughout the surgical care trajectory.

For patients with spinal metastases, spouses are not only caregivers but also important partners in the patient’s illness experience and emotional adjustment. Previous studies have shown that, in the context of advanced cancer, patients and their spouses exhibit substantial interdependence in emotional responses, illness-related communication, caregiving burden, and coping strategies (6, 7). Spouses’ negative emotions and caregiving stress may interact with patients’ psychological stress, communication patterns, and quality of life. In this context, supportive interventions targeting only the individual patient may be insufficient to fully improve perioperative psychosocial adjustment. Therefore, using the patient–spouse dyad as the basic unit of intervention better reflects the real-world care structure of patients with cancer (8, 9).

Although perioperative communication is widely emphasized by healthcare professionals in clinical practice, routine communication may depend on individual clinician experience, may lack standardized content and timing, and may not systematically involve spouses as active co-participants. For patients undergoing surgery for spinal metastases, disease-related threats, pain, functional uncertainty, and post-discharge caregiving tasks are often jointly faced by patients and their spouses (10–12). Guided by this dyadic interdependence perspective, the intervention was designed to target shared appraisal, emotional regulation, communication, and coordinated coping across the perioperative trajectory rather than either member of the couple in isolation. The primary clinical outcome was patient emotional distress, while spouse distress, dyadic coping, and spouse caregiving burden were evaluated as secondary outcomes. The purpose of this study was to evaluate a sequential, brief, and reproducible patient–spouse dyadic psychological intervention using a randomized controlled design and to provide an operationally feasible approach to perioperative psychosocial nursing care for patients with spinal metastases.

## 2 Materials and methods

### 2.1 Study participants

This study adopted a prospective randomized controlled trial design. A total of 92 patients with spinal metastases who underwent surgical treatment and their spouses were recruited from the Pre-admission Preparation Center and the Department of Spinal Degeneration and Tumors of Weifang People’s Hospital between November 2024 and January 2026. The inclusion criteria for patients were as follows: (a) pathological or radiological diagnosis of spinal metastasis; (b) both members of the dyad aged 18–75 years; (c) expected survival time of at least 6 months; and (d) written informed consent provided by both the patient and the spouse. The exclusion criteria were as follows: (a) severe mental illness, such as schizophrenia or bipolar disorder; (b) critical condition or marked perioperative changes that prevented completion of the study procedures; (c) severe cardiopulmonary insufficiency or hearing impairment; and (d) primary spinal tumors. The dropout criteria were as follows: (a) poor adherence to the intervention; (b) temporary change of the surgical procedure to a non-open surgery; and (c) voluntary withdrawal from the study. The inclusion criteria for spouses were as follows: (a) being the patient’s legal spouse or long-term stable partner, undertaking the main caregiving responsibilities during the perioperative period, and being able to participate jointly in the intervention and assessments; (b) aged 18–75 years, with basic language communication ability and questionnaire comprehension ability, and able to cooperate with data collection; and (c) providing informed consent and voluntarily participating in the study. The exclusion criteria for spouses were as follows: (a) severe mental illness or obvious cognitive impairment that prevented participation in communication, intervention, and assessment; (b) severe hearing, speech, or visual impairment affecting communication or questionnaire completion; (c) severe physical illness or unstable current health status that prevented continued participation in the study; and (d) not being the main perioperative caregiver of the patient or being unable to participate in the main intervention and follow-up according to the study requirements. No formal *a priori* sample-size calculation was performed; enrollment was determined pragmatically by the number of eligible patient–spouse dyads recruited during the study period. The protocol did not specify depressive or anxiety disorders as separate exclusion criteria.

### 2.2 Study design and ethics

This was a single-center, prospective, randomized controlled study. The patient–spouse dyad, consisting of a patient with spinal metastasis and the patient’s primary spouse caregiver, was the unit of randomization and intervention. Eligible dyads were randomly assigned to either the intervention group or the control group. The control group received routine perioperative nursing care, whereas the intervention group received a sequential patient–spouse dyadic psychological intervention in addition to routine nursing care. Data were collected 1–3 days before surgery, 1–3 days after surgery, on the day of discharge, and at the post-discharge follow-up. Patient- and spouse-reported outcomes were analyzed separately in the primary role-specific models, with supplementary joint dyad-aware models used to account for correlation between members of the same dyad. This study was reviewed and approved by the Medical Ethics Committee of Weifang People’s Hospital, with the ethics approval number KYLL20241028-18. The study strictly followed the principles of informed consent, voluntary participation, privacy protection, beneficence, and non-maleficence. Before the start of the study, the researchers provided patients and their spouses with detailed information regarding the study purpose, content, implementation procedures, potential benefits, and possible discomforts.

They were clearly informed that they had the right to withdraw from the study at any stage without any condition and that withdrawal would not affect their routine treatment or nursing care. Written informed consent was obtained from both patients and spouses after they had been fully informed and had voluntarily agreed to participate. During the study, all data were anonymized using coded identifiers and were used only for scientific analysis. The personal privacy of all participants was strictly protected. For patients or spouses who experienced obvious emotional fluctuation or worsening psychological distress during the study, the research team conducted timely assessments and provided further support or referral to relevant professionals when necessary, to ensure the physical and psychological safety of the participants.

### 2.3 Randomization

Randomization was performed using a random number table. After completion of the baseline assessment at T0, group allocation was conducted by opening sealed envelopes. A researcher who was not involved in participant recruitment, intervention delivery, or outcome assessment generated the random allocation sequence at a 1:1 ratio and placed the group assignments into sequentially numbered, opaque, sealed envelopes. After the participants completed the baseline assessment, a designated staff member opened the envelopes sequentially according to the order of enrollment to determine group assignment, thereby ensuring randomization and allocation concealment. Given the behavioral nature of the intervention, blinding of participants and intervention providers was not feasible. However, outcome data collection and statistical analysis were performed, whenever possible, by researchers who were not involved in intervention delivery, in order to reduce measurement and analytical bias.

### 2.4 Intervention group

The intervention was organized around a dyadic interdependence framework in which stress appraisal, emotional regulation, communication, and coping were treated as linked processes within the couple. Threat identification targeted shared appraisal of the most salient perioperative stressors; brief breathing or body-orientation exercises targeted immediate emotional arousal; expectation alignment targeted discrepant patient–spouse expectations; the “pause–name–respond” sequence and three-sentence communication framework targeted emotion recognition and explicit supportive communication; and the post-discharge dyadic coping plan translated these skills into coordinated problem solving and help-seeking. The expected mechanism was therefore improvement in how the dyad jointly identified, communicated, and managed stress rather than an effect operating primarily through either the patient or the spouse alone.

The intervention protocol emphasized a sequential process rather than a single episode of supportive communication. During the preoperative stage, patients and spouses mainly faced perceived threats related to surgical risk, persistent pain, changes in neurological function, and disease progression. In the early postoperative stage, emotional distress was more closely related to pain, physical weakness, restricted activity, and uncertainty regarding recovery. During discharge preparation and the transition to home care, stress gradually shifted toward family caregiving arrangements, identification of abnormal symptoms, communication with healthcare professionals, and dyadic coping with long-term disease uncertainty. Therefore, the intervention content was arranged in stages according to changes in perioperative tasks, so that each intervention session corresponded to the primary psychosocial needs of the patient–spouse dyad at a specific time point.

Specifically, the first preoperative intervention focused on threat identification and emotional stabilization. The research nurse guided patients and spouses to express their most important current concerns separately and helped them distinguish between “currently manageable problems” and “problems that remain uncertain at present.” This process was intended to reduce generalized and catastrophizing worry. Brief breathing relaxation or body-orientation exercises were also used to reduce immediate emotional arousal. The second preoperative intervention focused on expectation alignment and role preparation. By explaining the goals and limitations of surgery and discussing realistic short-term postoperative goals, the research nurse helped patients and spouses develop more consistent perioperative expectations and clarify the spouse’s main roles in postoperative observation, communication, and assistance. The third intervention was delivered 24–48 h after surgery and focused on the relationship between symptoms and emotions. The research nurse assisted patients in identifying the symptoms that most strongly affected their emotions and the corresponding emotional responses. The nurse also helped spouses recognize their own common reactions and taught the three-step method of “pause–name–respond,” enabling both members of the dyad to first identify the problem and then provide more targeted responses when facing pain, anxiety, or caregiving stress. The fourth intervention was delivered 3–5 days after surgery and focused on dyadic communication and joint problem solving. The research nurse guided both members of the dyad to identify common ineffective postoperative communication patterns and learn a “three-sentence communication” framework: expressing the most uncomfortable current problem, explaining the support most needed at present, and jointly determining what should be done first. Based on this framework, a 24-h short-term goal was developed. The fifth intervention was delivered before discharge and focused on preparation for the transition to home care and development of a dyadic coping plan. The research nurse guided patients and spouses to review difficult situations encountered during hospitalization and the coping strategies that had been effective. Together, they developed a two-week post-discharge coping plan, including symptom observation, identification of abnormal conditions, activity arrangements, family role division, communication agreements, and help-seeking strategies. Two reinforcement follow-ups were conducted on days 7 and 14 after discharge. These follow-ups were mainly used to monitor the implementation of the dyadic coping plan, identify new emotional distress or caregiving stress, reinforce the communication and coping skills learned earlier, and assess the need for referral to professional support when necessary.

In addition to outlining the intervention framework in the main text, the details of the intervention were further presented in Supplementary Tables. Supplementary Table 1 shows the stage division, implementation frequency, timing, and core objectives of the sequential patient–spouse dyadic psychological intervention. Supplementary Table 2 presents the specific operational content, main tasks, and expected outputs of each intervention session. Supplementary Table 3 presents the materials used in intervention delivery, standardized prompts, quality control procedures, and key points for managing deviations.

### 2.5 Control group

The control group received routine perioperative nursing care delivered by responsible nurses according to the standard nursing procedures of the department. The main components included admission assessment and health education, preoperative preparation guidance, condition monitoring, pain assessment and management, basic postoperative nursing care, functional exercise guidance, and discharge education. Spouses received necessary education on accompanying care and caregiving guidance. Both groups completed the same scheduled outcome assessments. The control group did not receive the five structured dyadic sessions or the two scheduled reinforcement follow-ups that constituted the study intervention.

### 2.6 Outcome measures

The outcome measures included the following: (a) General information questionnaire. Basic information was collected, including sex, age, marital status, educational level, place of residence, and previous treatment history. (b) Emotional distress. Patient emotional distress was the primary outcome and was measured using the Distress Thermometer (DT) (13, 14). The DT is a rapid distress screening tool widely used in oncology. It uses a 0–10 scoring scale, with higher scores indicating greater emotional distress. Spouse emotional distress measured using the DT was analyzed as a secondary outcome. The Problem List (PL) was used as a supplementary assessment tool to help identify the main sources of distress and was not included as an outcome measure in the statistical analysis. (c) Patient–spouse dyadic coping. Patient and spouse dyadic coping scores were key secondary outcomes and were measured using the Dyadic Coping Inventory (DCI) (15, 16). The DCI evaluates dimensions including stress communication, supportive coping, delegated coping, and common dyadic coping; higher total scores indicate better dyadic coping. (d) Spouse caregiving burden. Spouse caregiving burden was an additional secondary outcome and was measured using the Zarit Burden Interview (ZBI) (17, 18), with higher scores indicating greater caregiving burden.

Data were collected from patients and spouses 1–3 days before surgery, 1–3 days after surgery, before discharge, and 2 weeks after discharge. The assessment 1–3 days before surgery was defined as T0 and was completed after informed consent and enrollment but before the first intervention, in order to evaluate the baseline status before intervention. After completion of the T0 assessment, the intervention group began the first session of the sequential patient–spouse dyadic psychological intervention. The assessment 1–3 days after surgery was defined as T1, the pre-discharge assessment as T2, and the two-week post-discharge follow-up as T3.

Emotional distress in patients and spouses was measured using the DT at T0, T1, T2, and T3. Patient–spouse dyadic coping was measured using the DCI at T0, T2, and T3. Spouse caregiving burden was measured using the ZBI at T0, T2, and T3. Within each assessment window, each participant was assessed only once. The assessment was conducted at a time when the patient’s condition was relatively stable and the participant was able to cooperate with questionnaire completion.

### 2.7 Statistical analysis

Continuous variables were summarized as mean ± standard deviation, and categorical variables as frequencies and percentages. Baseline continuous variables were compared using independent-samples *t* tests, and categorical variables using the χ^2^ test or Fisher’s exact test as appropriate. All statistical tests were two-sided, with *p* < 0.05 considered statistically significant.

Longitudinal postbaseline outcomes were analyzed using baseline-adjusted linear mixed-effects models. T0 was treated as a baseline covariate and was not included as a repeated outcome. For DT, the repeated outcomes were T1, T2, and T3; for DCI and ZBI, the repeated outcomes were T2 and T3. Time was modeled as a categorical factor. In each role-specific model, group, time, and the group-by-time interaction were included as fixed effects together with the corresponding T0 score, and participant ID was included as a random intercept to account for within-participant repeated measurements. Group and time were effect-coded so that the group main effect represented the adjusted average between-group difference across the postbaseline assessment period. Models were estimated by restricted maximum likelihood (REML), and fixed effects were evaluated using Wald χ^2^ tests. Time-specific adjusted between-group contrasts were derived from the fitted models with 95% confidence intervals; *p* values within each outcome-specific contrast family were adjusted using the Holm method. For DT, standardized between-group effect sizes were additionally summarized using Hedges’ g with 95% confidence intervals. An exploratory responder analysis compared the proportions of participants with DT < 4 at each postbaseline time point using Fisher’s exact test.

Because patient and spouse observations from the same couple are not independent, supplementary dyad-aware mixed-effects models were fitted for DT and DCI by jointly stacking patient and spouse observations. These models included group, role (patient versus spouse), time, all corresponding two-way interactions, and the group-by-role-by-time interaction as fixed effects, together with the role-specific T0 score as a baseline covariate. A dyad-level random intercept and random role slope were included, with an unstructured covariance matrix for these random effects, thereby allowing repeated observations within each participant and correlation between members of the same dyad to be represented in the model. Actor–Partner Interdependence Model mediation pathways were not estimated because actor and partner pathways were not prespecified trial estimands. Exploratory sensitivity analyses were also performed using verified individual-level covariates. Patient models were additionally adjusted for age, pain severity, ECOG performance status, and surgical approach; spouse models were additionally adjusted for daily caregiving time and cohabitation status. These covariate sets were not prespecified and the sensitivity analyses were therefore considered exploratory. Analyses were performed in Python 3.13.5 using statsmodels 0.14.6; mixed models were estimated by REML.

## 3 Results

### 3.1 Comparison of baseline characteristics

A total of 92 patient–spouse dyads were initially screened. Four dyads were excluded because they did not meet the inclusion criteria or did not complete the baseline assessment. The remaining 88 dyads completed randomization and were included in the analysis, with 44 dyads in each group. No post-randomization outcome data were missing through T3 in the analytic dataset. The main baseline characteristics were generally comparable between the two groups (Table 1). At baseline, the mean DT score was 5.37 in patients and 5.48 in spouses, both above the clinical screening threshold of DT ≥ 4. Baseline DCI and ZBI scores are presented descriptively in Table 1 without assigning a clinical category that was not prespecified for this study (see Figure 1).

**Table 1:** Comparison of baseline characteristics of patients and spouses between the two groups.

Variable	Control group (*n* = 44)	Intervention group (*n* = 44)	*p* value
Patient characteristics			
Age, years	55.09 ± 11.88	58.34 ± 13.12	0.227
BMI, kg/m^2^	24.37 ± 2.17	24.13 ± 2.93	0.667
Pain severity	5.41 ± 1.09	5.61 ± 1.32	0.428
Baseline DT score of patients	5.55 ± 1.15	5.20 ± 1.00	0.142
Baseline DCI score of patients	103.09 ± 11.07	105.20 ± 10.14	0.353
Sex			1.000
Male	22 (50.0%)	22 (50.0%)	
Female	22 (50.0%)	22 (50.0%)	
Educational level			0.874
Primary school or below	3 (6.8%)	4 (9.1%)	
Middle school	34 (77.3%)	32 (72.7%)	
College or above	7 (15.9%)	8 (18.2%)	
Previous radiotherapy or chemotherapy			0.515
Yes	20 (45.5%)	16 (36.4%)	
No	24 (54.5%)	28 (63.6%)	
Place of residence			0.906
Urban area	15 (34.1%)	17 (38.6%)	
Township	13 (29.5%)	12 (27.3%)	
Rural area	16 (36.4%)	15 (34.1%)	
ECOG performance status			0.967
0	4 (9.1%)	5 (11.4%)	
1	18 (40.9%)	19 (43.2%)	
2	17 (38.6%)	15 (34.1%)	
3	5 (11.4%)	5 (11.4%)	
Surgical approach			0.916
1 (PKP)	24 (54.5%)	24 (54.5%)	
2 (Tumor resection, decompression, bone grafting, and fusion)	10 (22.7%)	12 (27.3%)	
3 (Posterior decompression and pedicle screw internal fixation)	7 (15.9%)	5 (11.4%)	
4 (Vertebral body resection and reconstruction)	3 (6.8%)	3 (6.8%)	
Spouse characteristics			
Age, years	55.16 ± 11.88	58.14 ± 12.57	0.257
Baseline DT score of spouses	5.61 ± 1.06	5.34 ± 0.96	0.210
Baseline DCI score of spouses	108.07 ± 9.90	108.91 ± 10.22	0.696
Baseline ZBI score of spouses	35.09 ± 6.10	36.59 ± 5.80	0.240
Sex			1.000
Male	22 (50.0%)	22 (50.0%)	
Female	22 (50.0%)	22 (50.0%)	
Educational level			0.589
Primary school or below	3 (6.8%)	3 (6.8%)	
Middle school	33 (75.0%)	29 (65.9%)	
College or above	8 (18.2%)	12 (27.3%)	
Occupational status			0.155
Employed	10 (22.7%)	6 (13.6%)	
Retired	16 (36.4%)	11 (25.0%)	
No fixed occupation	18 (40.9%)	27 (61.4%)	
Cohabitation status			0.501
Living together	41 (93.2%)	43 (97.7%)	
Not living together	2 (4.5%)	1 (2.3%)	
Living together part of the time	1 (2.3%)	0 (0.0%)	
Daily caregiving time			0.798
< 4 h	1 (2.3%)	0 (0.0%)	
4–8 h	1 (2.3%)	1 (2.3%)	
8–12 h	6 (13.6%)	6 (13.6%)	
> 12 h	36 (81.8%)	37 (84.1%)	

Continuous variables are presented as mean ± standard deviation, and categorical variables are presented as *n* (%). BMI, body mass index; DT, Distress Thermometer; DCI, Dyadic Coping Inventory; ZBI, Zarit Burden Interview; ECOG, Eastern Cooperative Oncology Group; PKP, percutaneous kyphoplasty.

**Figure 1:**
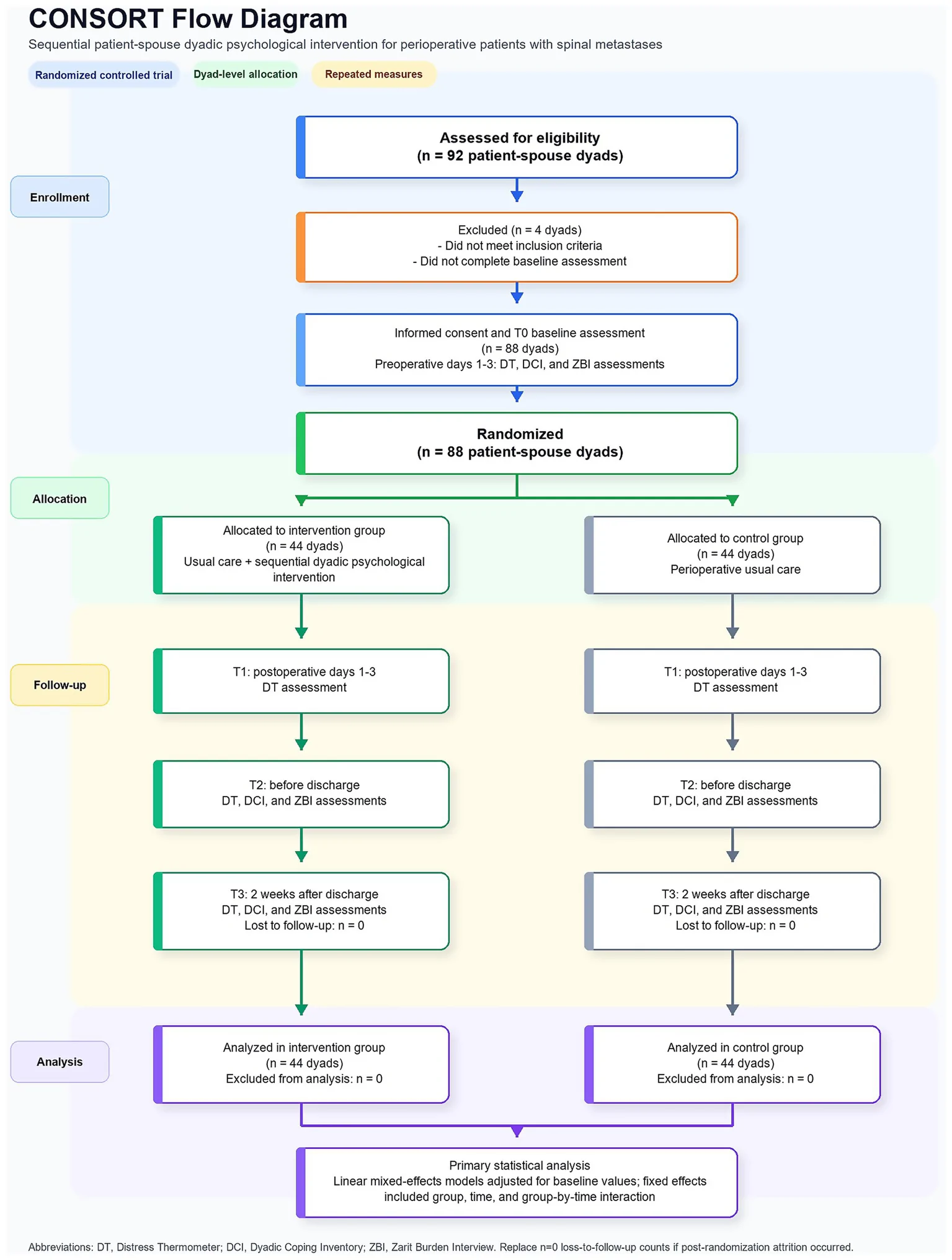
CONSORT flow diagram.

### 3.2 Longitudinal changes in emotional distress among patients and spouses

The baseline-adjusted role-specific mixed-effects model results are shown in Table 2. For patient DT, the primary outcome, the overall group effect was significant (Wald χ^2^ = 7.05, df = 1, *p* = 0.008), corresponding to an adjusted average intervention-minus-control difference of −0.46 points (95% CI, −0.79 to −0.12) across T1–T3. The time effect was significant (Wald χ^2^ = 62.99, df = 2, *p* < 0.001), whereas the patient-specific group-by-time interaction was not (Wald χ^2^ = 4.15, df = 2, *p* = 0.125). For spouse DT, the group effect (Wald χ^2^ = 16.42, df = 1, *p* < 0.001) and time effect (Wald χ^2^ = 30.19, df = 2, *p* < 0.001) were significant, while the group-by-time interaction was not (Wald χ^2^ = 3.49, df = 2, *p* = 0.175). The adjusted average spouse DT difference was −0.62 points (95% CI, −0.92 to −0.32).

**Table 2:** Baseline-adjusted linear mixed-effects model results for DT scores in patients and spouses.

Outcome	Effect	Wald χ^2^	df	*p* value	Adjusted difference (95% CI)
Patient DT	Group	7.05	1	0.008	−0.46 (−0.79 to −0.12)
	Time	62.99	2	<0.001	
	Group × time	4.15	2	0.125	
Spouse DT	Group	16.42	1	<0.001	−0.62 (−0.92 to −0.32)
	Time	30.19	2	<0.001	
	Group × time	3.49	2	0.175	

T0 was included as the corresponding baseline covariate and was not modeled as a repeated outcome. Group and time were effect-coded. Negative adjusted differences indicate lower DT scores in the intervention group. Wald *χ*^2^ tests are reported for fixed effects. DT, distress thermometer; CI, confidence interval.

Time-specific adjusted comparisons are shown in Table 3. For patient DT, no between-group difference was observed at T1. The adjusted difference at T2 was −0.52 points (95% CI, −0.99 to −0.06), but it did not remain statistically significant after Holm correction (adjusted *p* = 0.055). At T3, patient DT was lower in the intervention group by 0.71 points (95% CI, −1.17 to −0.24; Holm-adjusted *p* = 0.009), with a standardized difference of Hedges’ g = −0.67 (95% CI, −1.10 to −0.24). For spouse DT, the adjusted differences were not significant at T1 but were significant at T2 (−0.68; 95% CI, −1.17 to −0.19; Holm-adjusted *p* = 0.014) and T3 (−0.91; 95% CI, −1.40 to −0.41; Holm-adjusted *p* < 0.001). The corresponding T3 standardized difference was g = −0.79 (95% CI, −1.23 to −0.36).

**Table 3:** Adjusted between-group comparisons and standardized effect sizes for DT scores.

Outcome	Time point	Adjusted difference	95% CI	Holm-adjusted P	Hedges’ g (95% CI)
Patient DT	T1	−0.14	−0.60 to 0.33	0.564	−0.18 (−0.60 to 0.24)
	T2	−0.52	−0.99 to −0.06	0.055	−0.52 (−0.94 to −0.09)
	T3	−0.71	−1.17 to −0.24	0.009	−0.67 (−1.10 to −0.24)
Spouse DT	T1	−0.27	−0.76 to 0.22	0.284	−0.31 (−0.73 to 0.11)
	T2	−0.68	−1.17 to −0.19	0.014	−0.61 (−1.04 to −0.18)
	T3	−0.91	−1.40 to −0.41	<0.001	−0.79 (−1.23 to −0.36)

Adjusted differences were estimated from baseline-adjusted mixed-effects models and are expressed as intervention minus control; negative values favor the intervention for DT. *p* values were adjusted within the patient and spouse time-contrast families using the Holm method. Hedges’ g is based on the observed group means and pooled standard deviation; negative values indicate lower distress in the intervention group. DT, distress thermometer; CI, confidence interval.

An exploratory responder analysis using DT < 4 showed that at T3, 7 of 44 patients (15.9%) in the intervention group and 2 of 44 (4.5%) in the control group were below the screening threshold (Fisher’s exact *p* = 0.157). Among spouses, the corresponding proportions were 5 of 44 (11.4%) and 1 of 44 (2.3%), respectively (*p* = 0.202). Thus, the direction favored the intervention, but the responder differences were not statistically significant.

In the supplementary joint dyad-aware DT model, the overall group effect was significant (Wald χ^2^ = 19.60, df = 1, *p* < 0.001), as were the time effect (Wald χ^2^ = 86.18, df = 2, *p* < 0.001) and group-by-time interaction (Wald χ^2^ = 7.49, df = 2, *p* = 0.024). The group-by-role-by-time interaction was not significant (Wald χ^2^ = 0.02, df = 2, *p* = 0.988), providing no evidence that the treatment-related temporal pattern differed between patients and spouses (see Figure 2).

**Figure 2:**
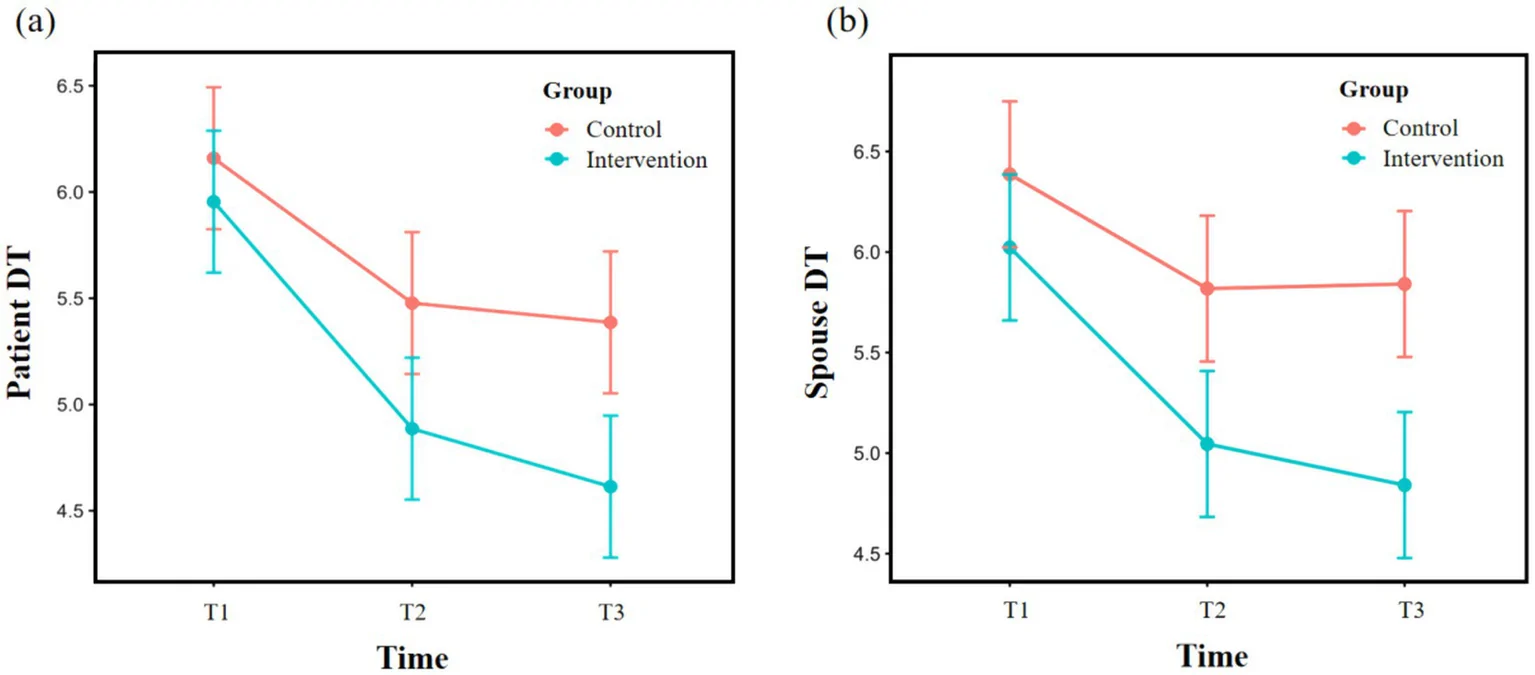
Trajectories of observed DT scores in patients and spouses across postoperative time points. **(a)** Patient DT; **(b)** spouse DT. Points represent observed group means and error bars indicate 95% confidence intervals. The *x*-axis shows T1 (early postoperative period), T2 (before discharge), and T3 (2 weeks after discharge).

### 3.3 Patient–spouse dyadic coping

The baseline-adjusted DCI models are shown in Table 4. Patient DCI scores were higher overall in the intervention group (Wald χ^2^ = 8.12, df = 1, *p* = 0.004), corresponding to an adjusted average difference of 5.50 points (95% CI, 1.72 to 9.28). The time effect (*p* = 0.146) and group-by-time interaction (*p* = 0.700) were not significant. Spouse DCI scores were also higher overall in the intervention group (Wald χ^2^ = 3.97, df = 1, *p* = 0.046; adjusted average difference, 3.33; 95% CI, 0.05 to 6.60), whereas the time effect (*p* = 0.183) and group-by-time interaction (*p* = 0.327) were not significant.

**Table 4:** Baseline-adjusted longitudinal analysis of DCI scores in patients and spouses.

Outcome	Group wald χ^2^ (*p*)	Time wald χ^2^ (*p*)	Group × time wald χ^2^ (*p*)
Patient DCI	8.12 (0.004)	2.11 (0.146)	0.15 (0.700)
Spouse DCI	3.97 (0.046)	1.77 (0.183)	0.96 (0.327)

T0 DCI was included as the baseline covariate. Group and time were effect-coded, and participant ID was included as a random intercept. Higher DCI scores indicate better dyadic coping. DCI, dyadic coping inventory.

Time-specific adjusted DCI comparisons are shown in Table 5. For patients, the T2 difference was 4.85 points (95% CI, −0.17 to 9.87; Holm-adjusted *p* = 0.058) and the T3 difference was 6.15 points (95% CI, 1.13 to 11.17; Holm-adjusted *p* = 0.033). For spouses, neither time-specific contrast remained statistically significant after Holm correction (T2 adjusted *p* = 0.429; T3 adjusted *p* = 0.067). In the joint dyad-aware DCI model, the group effect was significant (Wald χ^2^ = 12.20, df = 1, *p* < 0.001) and the time effect was modest (Wald χ^2^ = 3.90, df = 1, *p* = 0.048), whereas the group-by-time interaction (*p* = 0.342) and group-by-role-by-time interaction (*p* = 0.702) were not significant (see Figure 3).

**Table 5:** Adjusted between-group comparisons of DCI scores.

Outcome	Time point	Adjusted difference	95% CI	Raw *p*	Holm-adjusted *p*
Patient DCI	T2	4.85	−0.17 to 9.87	0.058	0.058
	T3	6.15	1.13 to 11.17	0.016	0.033
Spouse DCI	T2	1.80	−2.67 to 6.27	0.429	0.429
	T3	4.85	0.38 to 9.32	0.033	0.067

Adjusted differences were estimated from baseline-adjusted mixed-effects models and are expressed as intervention minus control; positive values indicate higher DCI scores in the intervention group. *p* values were adjusted within each role-specific time-contrast family using the Holm method. DCI, Dyadic Coping Inventory; CI, confidence interval.

**Figure 3:**
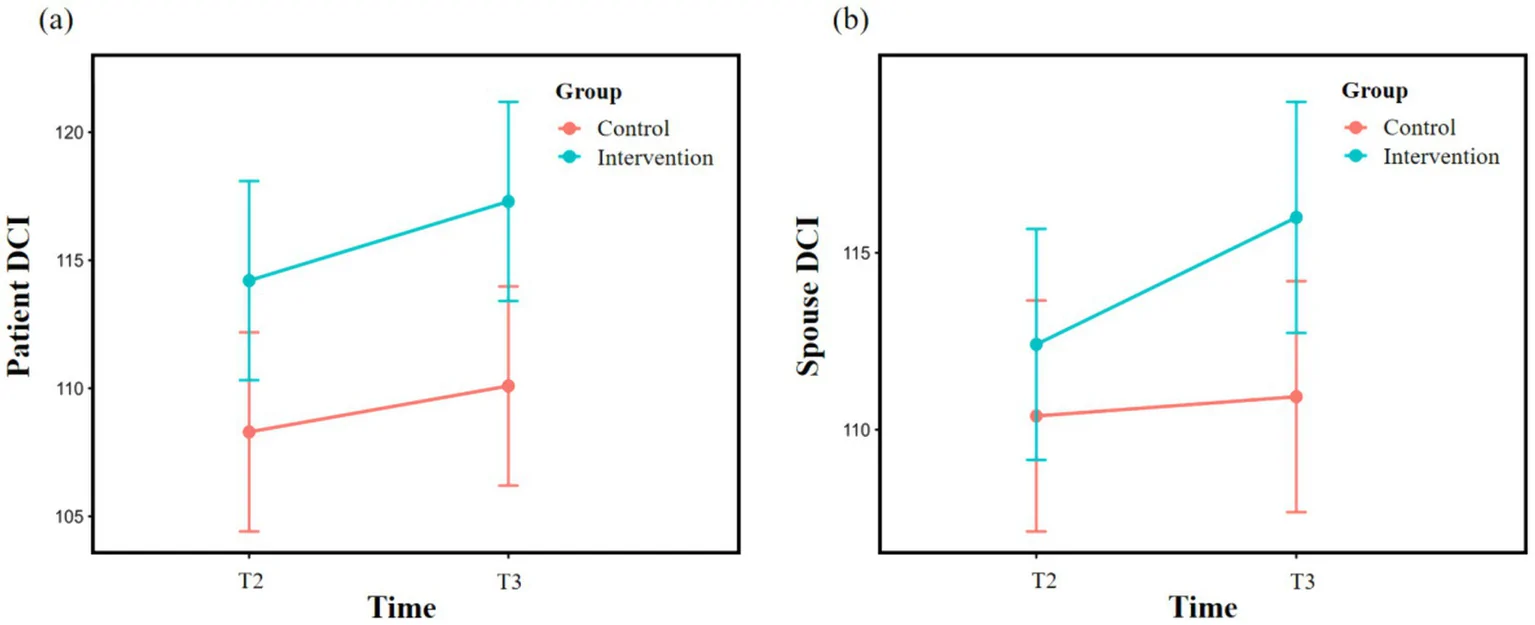
Trajectories of observed DCI scores in patients and spouses across postoperative time points. **(a)** Patient DCI; **(b)** spouse DCI. Points represent observed group means and error bars indicate 95% confidence intervals. The *x*-axis shows T2 (before discharge) and T3 (2 weeks after discharge).

### 3.4 Spouse caregiving burden

The baseline-adjusted ZBI model showed a significant overall group effect (Wald χ^2^ = 9.68, df = 1, *p* = 0.002), corresponding to an adjusted average intervention-minus-control difference of −3.24 points (95% CI, −5.28 to −1.20) across T2–T3. Neither the time effect (Wald χ^2^ = 0.38, df = 1, *p* = 0.536) nor the group-by-time interaction (Wald χ^2^ = 0.48, df = 1, *p* = 0.488) was significant. The time-specific adjusted difference was −2.60 points at T2 (95% CI, −5.32 to 0.12; Holm-adjusted *p* = 0.061) and −3.87 points at T3 (95% CI, −6.60 to −1.15; Holm-adjusted *p* = 0.011) (see Table 6).

**Table 6:** Baseline-adjusted longitudinal analysis of spouse ZBI scores.

Effect	Wald χ^2^ (df)	*p* value
Group	9.68 (1)	0.002
Time	0.38 (1)	0.536
Group × time	0.48 (1)	0.488

T0 ZBI was included as the baseline covariate. Group and time were effect-coded, and participant ID was included as a random intercept. Higher ZBI scores indicate greater caregiving burden. ZBI, Zarit Burden Interview.

### 3.5 Sensitivity analysis

Exploratory covariate-adjusted sensitivity analyses yielded conclusions consistent with the corresponding role-specific main models (Table 7). After additional adjustment for patient age, pain severity, ECOG performance status, and surgical approach, the overall group effect remained significant for patient DT (Wald χ^2^ = 5.60, *p* = 0.018) and patient DCI (Wald χ^2^ = 7.82, *p* = 0.005). After adjustment for daily caregiving time and cohabitation status, the group effects remained significant for spouse DT (Wald χ^2^ = 17.49, *p* < 0.001), spouse DCI (Wald χ^2^ = 4.52, *p* = 0.033), and spouse ZBI (Wald χ^2^ = 9.86, *p* = 0.002). The corresponding group-by-time interactions remained non-significant. Because these additional covariates were not prespecified, these analyses should be interpreted as exploratory.

**Table 7:** Exploratory sensitivity analyses after additional covariate adjustment.

Outcome	Additional covariates	Group Wald χ^2^ (P)	Time Wald χ^2^ (P)	Group × time Wald χ^2^ (P)	Conclusion
Patient DT	Age, pain severity, ECOG performance status, surgical approach	5.60 (0.018)	62.99 (<0.001)	4.15 (0.125)	Consistent
Spouse DT	Daily caregiving time, cohabitation status	17.49 (<0.001)	30.19 (<0.001)	3.49 (0.175)	Consistent
Patient DCI	Age, pain severity, ECOG performance status, surgical approach	7.82 (0.005)	2.11 (0.146)	0.15 (0.700)	Consistent
Spouse DCI	Daily caregiving time, cohabitation status	4.52 (0.033)	1.77 (0.183)	0.96 (0.327)	Consistent
Spouse ZBI	Daily caregiving time, cohabitation status	9.86 (0.002)	0.38 (0.536)	0.48 (0.488)	Consistent

All models retained the corresponding T0 score as a baseline covariate. The additional covariate sets were not prespecified and these sensitivity analyses are exploratory. ECOG, Eastern Cooperative Oncology Group; DT, Distress Thermometer; DCI, Dyadic Coping Inventory; ZBI, Zarit Burden Interview.

## 4 Discussion

This randomized trial evaluated a sequential dyadic psychological intervention for patients undergoing surgery for spinal metastases and their spouses. In the primary role-specific analysis, patient DT scores were lower overall in the intervention group across T1–T3, although the patient-specific group-by-time interaction was not statistically significant. The clearest patient-level difference was observed at 2 weeks after discharge, where the adjusted DT difference was −0.71 points and the standardized effect was moderate (Hedges’ g = −0.67). Spouse DT showed a similar pattern, with significant adjusted differences at T2 and T3 and a moderate standardized difference at T3. The supplementary joint dyad-aware analysis identified a significant group-by-time interaction for DT but no group-by-role-by-time interaction, suggesting a treatment-related temporal pattern at the dyad level without evidence that the pattern differed between patients and spouses. Importantly, mean DT scores remained above the screening threshold at T3 in both groups; the findings therefore indicate modest improvement in distress rather than resolution of clinically significant distress.

Perioperative emotional distress in patients with spinal metastases is characterized by a clear stage-specific pattern. In the early postoperative period, patients remain in an acute recovery phase involving pain, physical weakness, restricted activity, and treatment uncertainty, while spouses adapt rapidly to caregiving demands and disease-related stress (19–21). In the present data, DT scores increased from baseline to the early postoperative assessment in both groups before declining later in the perioperative trajectory. Acute symptom burden and surgical stress provide one plausible explanation for the absence of an early between-group difference. However, this is not the only possible explanation: by T2 the intervention group had received a greater cumulative intervention dose, and repeated administration of the DT across assessments may also have influenced response patterns. As patients moved toward discharge and home care, the sources of stress increasingly involved recovery uncertainty, caregiving tasks, and family role adjustment (22, 23). These factors should therefore be considered when interpreting the later divergence in DT scores.

The intervention used in this study was not limited to comforting communication. Instead, its components were sequenced around changing perioperative tasks: preoperative threat identification and expectation alignment, early postoperative symptom–emotion recognition, structured dyadic communication, short-term joint goals, and a two-week post-discharge coping plan. Materials including worry records, controllable/uncontrollable problem classification, symptom–emotion records, three-sentence communication, and post-discharge dyadic coping plans were used to operationalize these tasks. These components were intended to strengthen shared appraisal, communication, and coordinated problem solving rather than simply increase information delivery. The later between-group differences are therefore consistent with a cumulative, stage-linked intervention process, but the present trial cannot separate specific content effects from cumulative contact or expectancy effects (24, 25).

The DCI findings provide a more cautious picture of dyadic coping (26). Patient and spouse DCI scores were higher overall in the intervention group after baseline adjustment, but neither role-specific group-by-time interaction was significant. After Holm correction, the patient T3 contrast remained statistically significant, whereas the patient T2 and spouse T3 contrasts did not. Thus, the data support an overall between-group difference in dyadic coping during follow-up more strongly than a claim that the intervention changed the longitudinal trajectory of DCI scores.

These findings are compatible with the rationale for treating the patient–spouse dyad as the intervention unit rather than focusing solely on the individual patient (4, 27). For patients with spinal metastases, surgery cannot completely remove tumor-related threats, and pain, uncertainty regarding functional recovery, subsequent treatment arrangements, and changes in life roles continue to affect patients and their families. Spouses are important participants in symptom observation, emotional support, daily assistance, and medical communication (28–32). A dyadic intervention may therefore be clinically relevant because it addresses communication and coping processes that occur within the couple, although the present analyses do not establish a specific causal mediation pathway.

The supplementary dyad-aware analyses were added to reflect the non-independence of patient and spouse observations within each couple. For DT, the significant group-by-time interaction in the joint model and the absence of a group-by-role-by-time interaction suggest that the treatment-related temporal pattern was broadly similar across the two roles. For DCI, the joint model showed an overall group difference but no significant group-by-time or three-way interaction. These findings are consistent with the broader literature emphasizing reciprocal patient–caregiver processes in cancer (33, 34), but they should not be interpreted as evidence of specific actor or partner effects because Actor–Partner Interdependence Model pathways were not prespecified or formally tested. Future studies designed specifically for actor–partner mechanisms and with longer follow-up would be valuable (35, 36).

For spouse caregiving burden, the baseline-adjusted analysis showed a lower overall ZBI score in the intervention group, whereas the time effect and group-by-time interaction were not significant. The adjusted difference was clearest at T3. This pattern suggests a difference in perceived caregiving burden during the observation period but does not demonstrate that the intervention altered the trajectory of burden over time.

Caregiving burden is influenced by factors beyond emotional communication, including patient function, disease progression, surgical complexity, family resources, economic pressure, and caregiving time (37, 38). More than 80% of spouses in both groups reported more than 12 h of daily caregiving at baseline, but caregiving time was not repeatedly measured. The present study therefore cannot determine whether objective caregiving load remained constant while perceived burden differed between groups. Any interpretation that the intervention reduced subjective burden without changing objective workload should be considered provisional (39, 40).

This study has several limitations. First, it was conducted at a single center with a limited sample size, and no formal *a priori* sample-size calculation was performed; the precision and generalizability of the estimates therefore require confirmation in larger multicenter studies. Second, the trial was not prospectively registered, which limits independent verification of the original outcome hierarchy and analysis plan. Third, because this was a behavioral intervention, participants and intervention providers could not be blinded. In addition, the intervention arm received substantially more structured contact than the usual-care arm, and no dose-matched attention control was used; nonspecific effects of attention, therapeutic alliance, or expectancy cannot therefore be separated from the specific dyadic intervention content. Fourth, follow-up ended 2 weeks after discharge, so the persistence of distress reduction and dyadic coping changes beyond the acute transition remains unknown; the rising DCI pattern at T3 further supports the need for longer follow-up. Fifth, the analyses used DCI total scores and did not evaluate subscale-specific intervention effects or prespecified actor–partner mediation pathways. Finally, the intervention was developed within a Chinese hospital and family-caregiving context. Spousal caregiving norms, prognostic communication, and family participation in medical decision-making vary across health systems, so the intervention materials may require cultural adaptation rather than direct translation before use in other settings.

## Data Availability

The original contributions presented in the study are included in the article/Supplementary material, further inquiries can be directed to the corresponding author.
